# N-terminus of Flagellin Fused to an Antigen Improves Vaccine Efficacy against *Pasteurella Multocida* Infection in Chickens

**DOI:** 10.3390/vaccines8020283

**Published:** 2020-06-06

**Authors:** Thu-Dung Doan, Hsian-Yu Wang, Guan-Ming Ke, Li-Ting Cheng

**Affiliations:** 1 International Degree Program of Animal Vaccine Technology, International College, National Pingtung University of Science and Technology, 1, Shuefu Road, Neipu, Pingtung 91201, Taiwan; dung50biotech@gmail.com; 2 Graduate Institute of Animal Vaccine Technology, College of Veterinary Medicine, National Pingtung University of Science and Technology, 1, Shuefu Road, Neipu, Pingtung 91201, Taiwan; hyw@g4e.npust.edu.tw (H.-Y.W.); kegm@mail.npust.edu.tw (G.-M.K.)

**Keywords:** flagellin, adjuvant, N-terminus, chimeric protein, *Pasteurella multocida*, lipoprotein E

## Abstract

Flagellin from bacteria elicits a proinflammatory immune response and may act as a vaccine adjuvant. In this study, we evaluated the adjuvant effect of the N-terminus of flagellin (residues 1–99) when linked to an antigen (a truncated, conserved domain of lipoprotein E of *Pasteurella multocida*). Immunization of chickens with the antigen-adjuvant chimeric protein showed that the N-terminus of flagellin accelerated the antibody response and enhanced the cellular immunity (CD8^+^ T cell expansion). Stimulation of peripheral blood mononuclear cells from vaccinated chickens showed both T_H_1 (IFN-γ and IL-12) and T_H_2 (IL-4)-type cytokine gene expressions. In a challenge test, the N-terminus of flagellin increased the survival rate to 75%, compared to 25% in the antigen-only group. In conclusion, our study found that the N-terminus of flagellin can increase the immune response and enhance vaccine protection.

## 1. Introduction

Flagellin is the major structural protein of the bacterial flagellum, a whip-like structure responsible for the locomotion of flagellated bacteria [1,2]. Since the flagellum is essential to the bacteria, host immune systems have evolved to recognize flagellin as a sign of infection through Toll-like receptor 5 [3,4] or NOD-like receptor protein 4 inflammasome receptor NAIP5/6 [5]. When flagellin binds to TLR5 on sentinel cells, MyD88-dependent signaling is induced to activate the proinflammatory transcription factor nuclear factor-κB (NF-κB), resulting in the activation of innate and subsequent adaptive immunity [6,7]. Therefore, flagellin may be applied as a vaccine adjuvant to enhance the immune response.

Structural and functional studies have identified domains of flagellin critical for immune activation. Flagellin of *Salmonella* Typhimurium (*S*. Typhimurium) contains four domains, D0, D1, D2 and D3, arranged in a boomerang-like structure [8,9]. When flagellin monomers polymerize to form the flagellum filament, D0 and D1 are embedded within the core of the filament, while D2 and D3 protrude from the surface. Comparative analysis of flagellins from different bacteria showed that D0 and D1 are highly conserved, whereas D2 and D3 show greater variability in sequence and structure [10,11]. Mutational analysis pointed to a region of 13 residues within D1 as the interaction site with TLR5 [3]. A hotspot containing a conserved arginine residue in D1 has been identified to interact with the leucine-rich repeat 9 (LRR9) loop of TLR5 [10]. In actual application, an engineered polypeptide drug (CBLB502) containing complete D0 and D1 was shown to retain full ability at activating NF-κB signaling through TLR5 [12]. Along with a number of other studies [13,14], extensive data demonstrate that D0 and D1 are important for TLR5 activation.

Within D0 and D1, further analyses indicated that the N-terminus of flagellin may be relatively more important for TLR5 activation than the C-terminus. In terms of protein sequence, the domains of flagellin are arranged as, starting at the N-terminus, D0-D1-D2-D3-D2-D1-D0. Therefore, the complete reconstruction of D0 and D1 requires sequences from both the N- and the C-termini of flagellin, complicating the protein design efforts. It is possible that both the N- and C-termini are not required for TLR5 activation. Deletion of the C-terminal D0 portion (residues 444–492) did not abrogate TLR5 recognition [3]. Studies found that, while the N-terminus (residues 79–117) can stimulate the production of both T_H_1- (IFN-γ) and T_H_2- (IL-4)-type cytokines, the C-terminus (residues 477–508) is incapable of inducing a T_H_2 response [15,16]. These results lead us to believe that the N-terminus may be sufficient for TLR5 activation and can be further tested as a vaccine adjuvant.

In this study, we evaluated the adjuvant effect of the N-terminus of flagellin in vivo using a subunit vaccine for fowl cholera, a bacterial disease caused by *Pasteurella multocida* (*P. multocida*) serotype A. For the subunit vaccine design, the N-terminus of flagellin (*n*FliC) was linked to a truncated, conserved region of *P. multocida* lipoprotein E (*t*plpE) via a glycine-serine (GS) linker. After vaccination, humoral and cellular immune responses elicited by the vaccines were evaluated and challenge tests performed.

## 2. Materials and Methods

### 2.1. Bacteria Strains

S. Typhimurium (ATCC 14028) was cultured in Tryptic Soy Broth at 37 °C. *P. multocida* A3 (ATCC 15742) and a virulent field isolate of *P. multocida A*, Chu01 were cultured in a brain–heart infusion broth at 37 °C. Chu01 was confirmed to be of serogroup A with PCR using primers for the hyaD-hyaC gene [17].

### 2.2. Plasmid Construction and Protein Expression of Antigen-Adjuvant Recombinant Proteins

To evaluate the adjuvant effect of *n*FliC, plasmids were constructed to express two recombinant proteins as subunit vaccines: (1) *n*FliC-*t*plpE, the N-terminus of flagellin fused to truncated *P. multocida* plpE, and (2) *t*plpE, truncated plpE only. For the cloning of *t*plpE, full-length plpE (hereafter referred to as plpE) was first cloned from the DNA of *P. multocida* serotype A3 (ATCC 15742) using primers listed in Table S1 and ligated into the vector pET32a (Novagen, Darmstadt, Germany). Subsequently, a conserved region within plpE (residues 26–86) of high antigenicity and hydrophilicity was identified using the ExPASy server (https://web.expasy.org/protparam/). Primers for *t*plpE (Table S1) were used for subcloning to obtain *t*plpE. For the cloning of *n*FliC, full-length FliC (hereafter referred to as FliC) was first cloned from the DNA of *S.* Typhimurium and inserted into pET32a before the subcloning of *n*FliC (residues 1–99) using primers for *n*FliC (Table S1). Finally, to create the *n*FliC-*t*plpE construct, chimeric polymerase chain reaction (PCR) was performed using *n*FliC and *t*plpE PCR products as templates and primers for *n*FliC-*t*plpE (Table S1). Note that *n*FliC was linked to the N-terminus of *t*plpE. Final constructs were inserted into pET32a, and sequencing was performed for reconfirmation.

To express (1) *n*FliC-*t*plpE and (2) *t*plpE, respective plasmid constructs were used to transform *Escherichia coli* BL21 (DE3) (Yeastern Biotech, Taipei, Taiwan) according to the manufacturer’s instructions. Protein expression was then induced with 1-mM isopropyl-b-D-galactopyranoside (IPTG; Sigma, Darmstadt, Germany) at 37 °C for 4 h. Cells were harvested, lysed in native lysis buffer (300-mM KCl, 50-mM KH2PO4 and 5-mM Imidazole) and sonicated. The soluble fraction was used for recombinant protein purification through the His-tag with Bio-scale Mini Profinity IMAC cartridges (1 mL) (Bio-Rad, Hercules, CA, USA) according to the manufacturer’s instructions. Expression levels of the recombinant proteins were determined by 12% sodium dodecyl sulfate-polyacrylamide gel electrophoresis (SDS-PAGE) analysis using BSA protein standards. To confirm the identity of the recombinant proteins, Western blot assay was performed. Briefly, after gel electrophoresis, proteins were transferred onto polyvinylidene difluoride (PVDF) membranes (Merck, Darmstadt, Germany). 6X-His Tag antibody solution (Gentex, Hsinchu, Taiwan) at 1:5000 dilution was used as the primary antibody, and goat anti-mouse antibody conjugated to HRP (Gentex, Taiwan) was used as the secondary antibody at 1:5000 dilution. Western Lightning PLUS (PerkinElmer, Waltham, MA, USA) was used for color development. Endotoxin levels of the purified proteins were confirmed to be less than 0.125 EU/mL with the ToxinSensorTM Chromogenic LAL Endotoxin Assay Kit (GenScript, Piscataway, NJ, USA).

### 2.3. Analysis of Proinflammatory Cytokine mRNA Levels

To examine the immunostimulatory effect of the recombinant proteins, peripheral blood mononuclear cells (PBMCs) from unvaccinated chickens (n = 3, five-week-old Brown Leghorns from a local farm) were collected and stimulated with FliC, *n*FliC-*t*plpE, *t*plpE or PBS as the negative control. To obtain PBMCs, blood samples were collected in tubes containing disodium ethylenediaminetetraacetic acid (EDTA). Ficoll-Paque (Amersham Biosciences, Piscataway, NJ, USA) was then added, and the mixture was centrifuged at 252× *g* for 40 min. PBMC-containing fraction was collected, and the cells were washed twice and resuspended in RPMI-1640 (Gibco Invitrogen, Carlsbad, CA, USA) supplemented with 5% fetal bovine serum (Gibco Invitrogen, Carlsbad, CA, USA) at 2 × 10^6^ cells/mL. Freshly prepared PBMCs (2 × 10^6^ cells/well) were then added to 24-well plates containing 10 μg/mL of the recombinant proteins for a 2 h incubation at 37 °C, 5% CO_2_. Total RNA was then extracted with the Total RNA Extraction Miniprep System (Viogene, Taipei, Taiwan) and complementary DNA (cDNA) synthesized using the Reverse Transcriptase Kit (Applied Biosystems, Foster, CA, USA). Real-time PCR was carried out in the SmartCycler I (Cepheid, Sunnyvale, CA, USA) with primers (Table S2) for proinflammatory cytokines (IL-1β, IL-6 and IL-8) and the housekeeping gene glyceraldehyde-3-phosphate dehydrogenase (GAPDH). Expression levels of the cytokine genes were normalized to that of the GAPDH gene and expressed as an n-fold increase or decreased relative to the PBS control.

### 2.4. Vaccine Preparation and Immunization

Three vaccine formulations were prepared: (1) *n*FliC-*t*plpE, (2) *t*plpE and (3) PBS as the control. Fifty micrograms/dose of each purified recombinant protein, along with PBS-only, were formulated with the water-in-oil adjuvant Montanide ISA71 (Seppic, Paris, France) in a 4:6 (aqueous: oil) ratio for a final injection volume of 0.2 mL per chicken.

For immunization, 24 five-week-old Brown Leghorns from a local farm were randomly assigned to three groups of eight for the three different vaccine formulations. Each chicken was immunized twice subcutaneously two weeks apart. For immune response analysis, whole blood was collected on days 0, 7, 14 and 28 post-vaccination from three chickens per vaccine group. All animal experimental protocols (NPUST-106-055) were approved by the Animal Care and Use Committee, National Pingtung University of Science and Technology (NPUST). The experiments were conducted based on the Ethical Rules and Law of NPUST.

### 2.5. Analysis of Humoral Immune Response

To determine the antibody response elicited by the vaccines, whole blood from immunized chickens was allowed to coagulate and then centrifuged at 700× *g* for 5 min to collect serum. Indirect enzyme-linked immunosorbent assay (ELISA) was carried out by coating plates with 50-ng/well purified plpE overnight at 4 °C. After washing and blocking, serum samples at 1:10,000 dilution were added as the primary antibody. Horseradish peroxidase (HRP)-conjugated anti-chicken IgG (Sigma, Carlsbad, CA, USA) at a 1:5000 dilution was used as the secondary antibody. The Peroxidase Kit (KPL, Gaithersburg, MD, USA) was used for color development, and optical density was read at 450 nm on the MultiskanTM FC microplate photometer (Thermo Fisher Scientific, Vantaa, Finland).

### 2.6. Analysis of Cellular Immune Response

The percentages of CD4^+^ and CD8^+^ T cells in the blood of immunized chickens were analyzed by flow cytometry to determine the cellular immune response elicited by the vaccines. PBMCs were collected from immunized chickens (days 14 and 28), as described in Section 2.3. For fluorescent labeling, PBMCs were washed and resuspended in PBS containing anti-CD4-PE or anti-CD8-FITC antibodies (Arigo, Hsinchu, Taiwan) for 45 min at 4 °C. Labeled cells were analyzed using the BD AccuriTM C6 flow cytometer (BD Biosciences, San Diego, CA, USA).

### 2.7. Analysis of T_H_1 and T_H_2 Type Cytokine mRNA Levels

Collected PBMCs from immunized chickens (day 28) were stimulated with 10 μg/mL of plpE to observe the types of cytokines produced. Stimulation experiment and real-time PCR were carried out as described Section 2.3 for T_H_1 (IFN-γ and IL12) and T_H_2-type cytokines (IL-4 and IL-10).

### 2.8. P. Multocida Challenge Test

On day 28 after vaccinations, chickens were challenged intramuscularly with 1.6 × 10^5^ CFU (10 LD_50_) of the highly virulent *P. multocida* strain Chu01. Chickens were monitored for clinical signs and survival rates recorded. Animal experimental protocols (NPUST-106-055) were approved by the Animal Care and Use Committee, National Pingtung University of Science and Technology (NPUST).

### 2.9. Statistical Analysis

Statistical analyses were performed using the IBM SPSS Statistics software version 22. One-way analysis of variance (ANOVA) and Tukey’s post hoc test were used for mean comparison for data from antibody response, cytokine mRNA levels and percentages of CD4^+^ and CD8^+^ T cells. Data are expressed as mean ± standard error of mean (SEM), and the significance level (*p*) was set at 0.05 for all immune analyses.

## 3. Results

### 3.1. An Antigen-Adjuvant Chimeric Protein Was Formulated as a Subunit Vaccine

**Figure 1:**
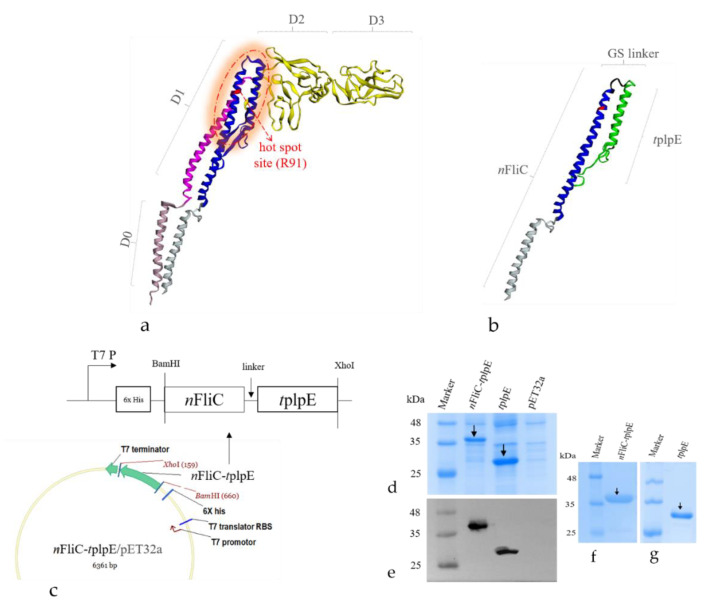
Chimeric protein (N-terminus of flagellin-truncated, conserved region of *P. multocida* lipoprotein E (nFliC-tplpE)) construction and protein analysis. (**a**) Previously solved 3D structure of the full-length flagellin (Protein Data Bank ID: 1UCU) as illustrated by the EzMol server is shown containing domains D0, D1, D2 and D3. (**b**) Predicted 3D structure of nFliC-tplpE using the Phyre2 server. (**c**) Cloning site for nFliC-tplpE into the pET32a expression vector. (**d**) SDS-PAGE and (**e)** Western blot analyses of expressed recombinant proteins. SDS-PAGE of purified (**f**) nFliC-tplpE and (**g**) tplpE proteins. GS: glycine-serine linker.

To evaluate whether *n*FliC may act as a vaccine adjuvant, a chimeric protein was constructed with a conserved domain of *P. multocida* plpE (*t*plpE) as the antigen and *n*FliC as the adjuvant (Figure 1). Structure of the *n*FliC-*t*plpE protein construct was predicted (Figure 1b). Protein expression was confirmed with SDS-PAGE (Figure 1d) and Western blot (Figure 1e) analyses, showing the target protein at 37 kDa (pET32a vector inserts a 20-kDa Trx-His-S-enterokinase tag). *n*FliC-*t*plpE was efficiently expressed in a soluble form at a concentration up to 800 mg/L, and the purified protein is shown (Figure 1f). To determine its immunostimulatory effect, *n*FliC-*t*plpE was first used to stimulate chicken PBMCs. For vaccination experiments, three vaccine formulations were prepared: (1) *n*FliC-*t*plpE, (2) *t*plpE and (3) PBS as the negative control. Chickens were vaccinated twice and blood collected for the immune response analysis.

### 3.2. N-terminus of Flagellin Enhanced the Proinflammatory Cytokine Gene Expression

**Figure 2:**
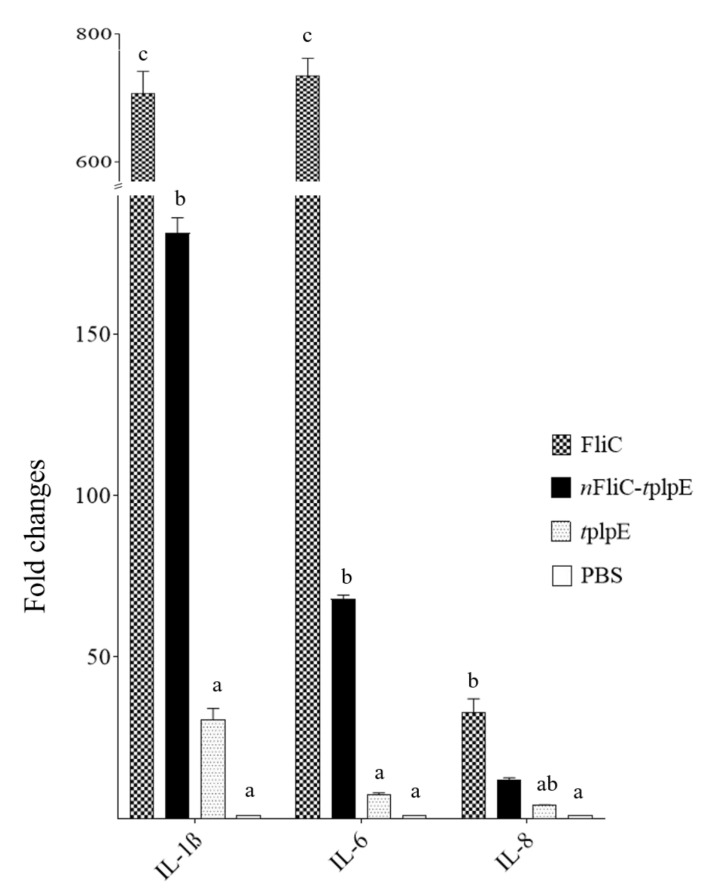
Proinflammatory cytokine gene expression. Peripheral blood mononuclear cells (PBMCs) from chickens (n = 3) were treated with 10 μg/mL of FliC, *n*FliC-*t*plpE, *t*plpE or PBS, and relative mRNA expression levels of IL-1β, IL-6 and IL-8 were determined. Data are presented as mean ± SEM. Different superscript letters indicate significant differences (*p* < 0.05) between treatment groups for each cytokine gene.

The ability of *n*FliC to enhance the proinflammatory cytokine gene expression was examined using chicken PBMCs. Compared to the *t*plpE construct, *n*FliC-*t*plpE significantly upregulated the expression levels of IL-1β, IL-6 and IL-8 (but not as high as those induced by FliC) (Figure 2). This confirmed that *n*FliC retained the immunostimulatory effect of FliC and may act as an effective adjuvant.

### 3.3. N-terminus of Flagellin Led to a Rapid Rise in Antibody Levels

**Figure 3:**
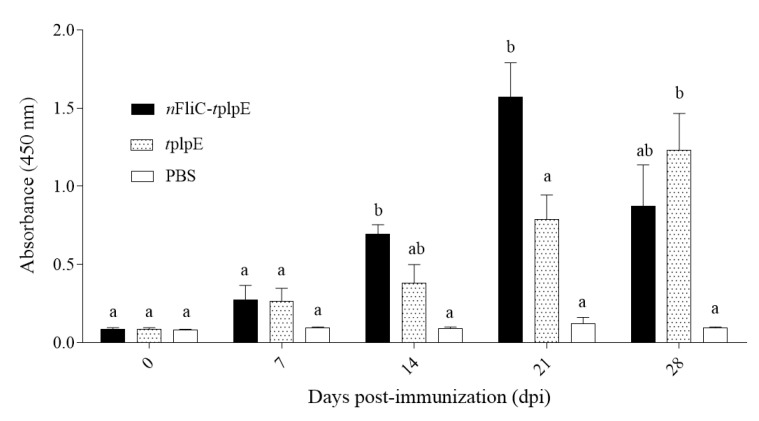
Antigen-specific antibodies of immunized chickens. Chickens (n = 3) were immunized twice with *n*FliC-*t*plpE, *t*plpE or PBS, and sera were analyzed by indirect ELISA using plpE as the coating antigen. Data are presented as mean ± SEM. Different superscript letters indicate significant differences (*p* < 0.05) between treatment groups at the same timepoint.

Serum antibody levels of immunized chickens were determined by indirect ELISA, with plpE as the coating antigen. On days 14 and 21 after vaccinations, *n*FliC-*t*plpE-vaccinated chickens showed significantly higher antibody levels than *t*plpE-vaccinated chickens (Figure 3), indicating that the addition of *n*FliC can accelerate the antibody response.

### 3.4. N-terminus of Flagellin Enhanced CD8^+^ T Cell Expansion

**Figure 4:**
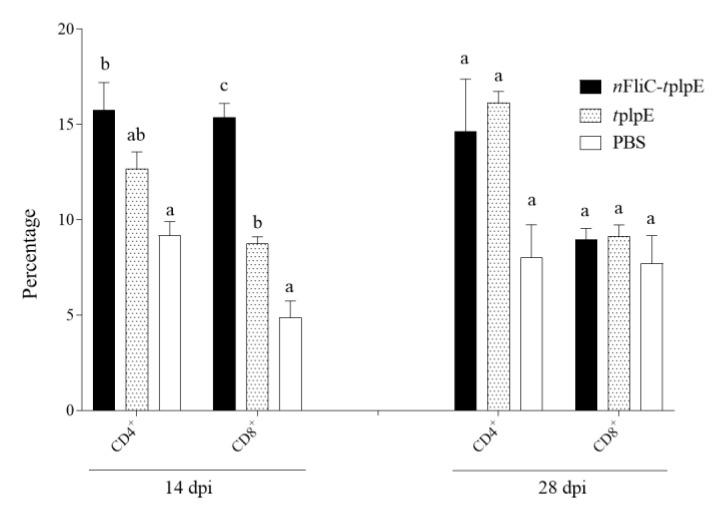
Percentages of CD4^+^ and CD8^+^ T cells in the PBMCs of immunized chickens. Chickens (n = 3) were immunized twice with *n*FliC-*t*plpE, *t*plpE or PBS, and isolated PBMCs were stained with anti-CD4 or -CD8 antibodies for flow cytometric analysis. Data are presented as mean ± SEM. Different superscript letters indicate significant differences (*p* < 0.05) between treatment groups at the same timepoint for CD4^+^ and CD8^+^ T cells.

The percentages of CD4^+^ and CD8^+^ T cells in PBMCs from immunized chickens were analyzed. As early as day 14 after immunization, CD4^+^ and CD8^+^ populations were seen expanded for both *n*FliC-*t*plpE and *t*plpE groups when compared to that of the PBS group (Figure 4). Further comparison showed that the CD8^+^ T cell percentage is significantly higher for the *n*FliC-*t*plpE group (15.4%) than the *t*plpE group (8.7%), showing that the addition of *n*FliC enhanced the cellular immune response early on. It is notable, however, that, by day 28, the CD8^+^ populations in the *n*FliC-*t*plpE group have contracted.

### 3.5. N-terminus of Flagellin Enhanced Gene Expressions of Both T_H_1 and T_H_2-Type Cytokines

**Figure 5:**
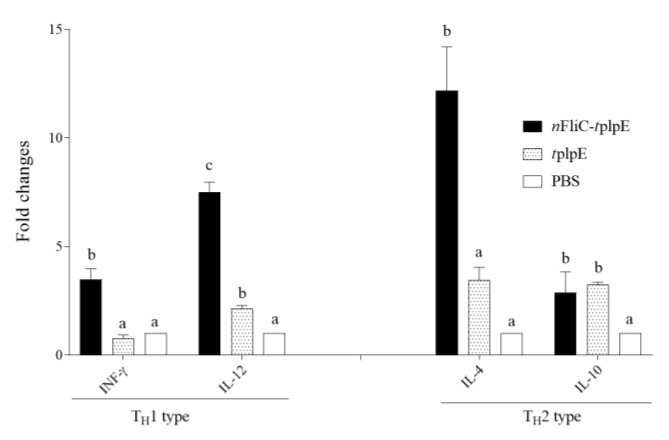
Cytokine gene expression of PBMCs from immunized chickens (28-day post-immunization (dpi)). Chickens (*n* = 3) were immunized twice with *n*FliC-*t*plpE, *t*plpE or PBS, and isolated PBMCs were stimulated with plpE. Relative mRNA expression levels of IFN-γ, IL-12, IL-4 and IL-10 were determined. Data are presented as mean ± SEM. Different superscript letters indicate significant differences (*p* < 0.05) between treatment groups for each cytokine.

To further explore the nature of the immune enhancement provided by *n*FliC, PBMCs from immunized chickens (28 days post-vaccination) were stimulated with plpE, and cytokine mRNA levels were quantitated. Results showed that *n*FliC enhanced both T_H_1 (IFN-γ and IL-12) and T_H_2-type (IL-4) cytokine levels (Figure 5), indicating a strong immune activation.

### 3.6. N-terminus of Flagellin Increased the Survival Rate to 75% in a Challenge Test

**Figure 6:**
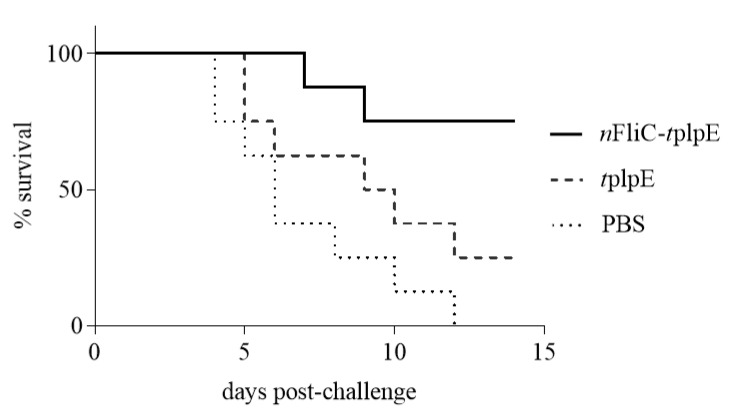
Survival rate of immunized chickens when challenged with *Pasteurella multocida*. Chickens (*n* = 8) were immunized twice with *n*FliC-*t*plpE, *t*plpE or PBS and challenged with 10 LD_50_ (1.6 × 10^5^ CFU/dose) *P. multocida*.

Vaccinated chickens (*n* = 8 per group) were challenged with the *P. multocida* strain Chu01. For the *t*plpE vaccine group, the survival rate was only 25% (Figure 6). For the *n*FliC-*t*plpE group, however, the survival rate increased to 75%, demonstrating that *n*FliC provided a significant boost to the protection.

## 4. Discussion

Functional studies of flagellin domains have established that D2 and D3 are dispensable for TLR5 activation, greatly improving the applicability of flagellin as an immune enhancer protein. With only D0 and D1 necessary for immune activation, the molecular size is reduced by half, improving protein expression and making recombinant antigen-adjuvant protein designs more feasible. Additionally, since most antibody responses to flagellin are directed at the decoy D3 domain, the deletion of D3 greatly reduced unwanted immunogenicity and toxicity [14]. Attempts to pinpoint the TLR5 activation domains of flagellin have allowed its redesign as a more “nimble” TLR5 agonist with less side effects.

Nevertheless, challenges remain in engineering D0/D1 as a vaccine adjuvant. To reconstruct D0/D1, the N- and C-terminal portions of D1 need to be reconnected. While the construction of the agonist drug CBLB502 required only a flexible linker sequence, insertion of an antigen to reconnect D1 may result in nonfunctional constructs. For example, attempts to replace the hypervariable region of flagellin with the vaccinia virus protein L1R resulted in antibodies that do not recognize native L1R [18]. A successful construct necessitates the proper folding of both the antigen and the adjuvant, which could be nontrivial in this more complicated design.

In attempts to make the D0/D1 construct more applicable to antigen-adjuvant constructs, we tested and found that the first 99 residues of the N-terminus of flagellin is sufficient for immune activation and vaccine protection enhancement. The crystal structure of flagellin bound to TLR5 demonstrated that three α-helices in the D1 domain bundle together in a rod and interact with the horseshoe-like external domain of TLR5 [11]. This interaction is critical for TLR5 binding. Two of the α-helices are located on the N-terminus of flagellin and one located on the C-terminus. The two N-terminal helices constitute a greater portion (~60%) of the binding interface than the C-terminal helix. The *n*FliC (residues 1–99) used in our study contains one of the N-terminal helices and, also, includes the absolutely conserved residue Arg91. Since an immune enhancement is observed for our *n*FliC-*t*plpE vaccine, the helix included in *n*FliC may play a more important role in TLR5 binding than the other two helices. On the other hand, if increased TLR5 binding is desired for the adjuvant design, the other two helices could be incorporated. Our data does show a lower gene expression level of proinflammatory cytokines for *n*FliC than full-length flagellin, indicating room for improvement.

Whereas D1 is important for TLR5 binding, D0 is essential for TLR5 signaling. Deletion of the D0 domain from CBLB502 resulted in an ~1000-fold drop in signaling efficiency [11], making D0 indispensable. Interestingly, a recent study demonstrated that flagellin lacking the C-terminal portion of D0 can still upregulate TLR5 signaling [19]. Therefore, the N-terminal portion of D0 of may be sufficient for TLR5 signaling. This observation is corroborated by the immune enhancement seen for our *n*FliC-*t*plpE construct, which contains the N-, but not C-terminal, D0 portion.

Overall, *n*FliC induced a cytokine expression profile similar to that of the full-length flagellin. Our in vitro PBMC stimulation experiment showed that *n*FliC elicited proinflammatory cytokines similar to those induced by FliC, albeit at a lower level. After the vaccination with *n*FliC-*t*plpE, isolated PBMCs produced mixed T_H_1 and T_H_2-type cytokine responses, which is consistent with observations from other studies using various constructs of flagellin [20]. The induction of both humoral and cellular immune responses generally indicates a more comprehensive immune activation; thus, the *n*FliC-*t*plpE construct may represent a promising vaccine design. It is interesting to note, however, that upon boosting with the *n*FliC-*t*plpE construct on day 14, both the antibody level and CD8^+^ T cells appeared to decrease by day 28. Further experimentation would help verify and clarify the mechanism underlying this observation.

## 5. Conclusions

We found *n*FliC capable of enhancing humoral and cellular immunity when fused to an antigen. Increased protection against a lethal challenge was also observed. Due to its reduced size, *n*FliC may also be an ideal adjuvant for multi-valent subunit vaccines.

## Supplementary Materials

The following are available online at https://www.mdpi.com/2076-393X/8/2/283/s1. Table S1: Primers for gene cloning and recombinant protein construction, Table S2: Primers for cytokine genes.
