# Supplementary material for: N-terminus of Flagellin Fused to an Antigen Improves Vaccine Efficacy against Pasteurella Multocida Infection in Chickens
Source: Vaccines (Basel). 2020 Jun 6;8(2):283. doi: 10.3390/vaccines8020283 (PMC7349934; doi:10.3390/vaccines8020283)
Supplement: Supplementary file 1 [file vaccines-08-00283-s001.pdf]

vaccines

**Table S1. Primers for gene cloning and recombinant protein construction.**

| Target Gene | | Sequence (5′-3′) | RE site | Gene-Length (bp) | DNA Templates |
|---|---|---|---|---|---|
| plpE | F | [1]*ggatcc*atgaaacaaatcgttttaaaaac | BamHI | 1008 | *ATCC 15742* |
| | R | *gaattc*ttattgtgcttggtgacttttttc | EcoRI | | |
| FliC | F | *ggatcc*atgaaaaagacaatcgtagc | BamHI | 1500 | *ATCC 14028* |
| | R | *gtcgac*ttagaagtgtacgcgtaaac | SalI | | |
| *t*plpE | F | [2]**ggcgggggcggcagc**ggtagcgctggaaatcg | - | 195 | *plpE* |
| | R | *ctcgag*aaaggaggattgttgactattt | XhoI | | |
| *n*FliC | F | *ggatcc*atggcacaagtcattaatacaaac | BamHI | 312 | *FliC* |
| | R | **gctgccgcccccgcc**agactgaaccgccagttc | - | | |
| *n*FliC-*t*plpE | F | *n*FliC F | BamHI | 495 | *t*plpE and *n*FliC |
| | R | *t*plpE R | XhoI | | |

[1] Italics in the primers represent restriction enzyme (RE) sites. [2] Bold fonts in the primers represent glycine-serine linkers.

Table S2. Primers for cytokine genes.

| Target Gene | | Sequence (5′-3′) | Length (bp) | Annealing Temp. (°C) | GenBank |
|---|---|---|---|---|---|
| IL-1β | F | tgggcatcaagggctaca | 244 | 55 | NM_204524.1 |
| | R | tcgggttggttggtgatg | | | |
| IL-6 | F | caaggtgacggaggaggac | 253 | 55 | JQ897539 |
| | R | tggcgaggagggatttct | | | |
| IL-8 | F | catcatgaagcattccatct | 204 | 50 | HM179639.1 |
| | R | cttcca agggatcttcattt | | | |
| IFN-γ | F | gacggtggacctattatt | 255 | 50 | HQ739082 |
| | R | ggctttgcgctggattc | | | |
| IL-12 | F | ccaagacctggagcacaccgaag | 163 | 61 | AY262752.1 |
| | R | gatccctggcctgcacagaga | | | |
| IL-4 | F | tgtgcccacgctgtgcttaca | 193 | 61 | AJ621249.1 |
| | R | cttgtggcagtgctggctctcc | | | |
| IL-10 | F | ctgcgcttctacacagatg | 427 | 55 | XM_025143715.1 |
| | R | ccgttctcatccatctgc | | | |
| GAPDH | F | tgctgcccagaacatcatcc | 142 | 55 | NM_204305 |
| | R | acggcaggtcaggtcaacaa | | | |

GAPDH: glyceraldehyde 3 phosphate dehydrogenase; IL: interleukin; IFN-γ: interferon gamma.
